# Pressure-Modulated Host*–*Guest Interactions Boost Effective Blue-Light Emission of MIL-140A Nanocrystals

**DOI:** 10.1007/s40820-025-01917-8

**Published:** 2025-09-15

**Authors:** Ting Zhang, Jiaju Liang, Ruidong Qiao, Binhao Yang, Kaiyan Yuan, Yixuan Wang, Chuang Liu, Zhaodong Liu, Xinyi Yang, Bo Zou

**Affiliations:** Synergetic Extreme Condition High-Pressure Science Center, State Key Laboratory of High Pressure and Superhard Materials, College of Physics, Jilin University, Changchun, 130012 People’s Republic of China

**Keywords:** Metal–organic framework nanocrystals, Blue-light emission, Host–guest interactions, Pressure treatment

## Abstract

Luminescent metal–organic frameworks (MOFs) have garnered significant attention due to their structural tunability and potential applications in solid-state lighting, bioimaging, sensing, anti-counterfeiting, and other fields. Nevertheless, due to the tendency of 1,4-benzenedicarboxylic acid (BDC) to rotate within the framework, MOFs composed of it exhibit significant non-radiative energy dissipation and thus impair the emissive properties. In this study, efficient luminescence of MIL-140A nanocrystals (NCs) with BDC rotors as ligands is achieved by pressure treatment strategy. Pressure treatment effectively modulates the pore structure of the framework, enhancing the interactions between the N, N-dimethylformamide guest molecules and the BDC ligands. The enhanced host*–*guest interaction contributes to the structural rigidity of the MOF, thereby suppressing the rotation-induced excited-state energy loss. As a result, the pressure-treated MIL-140A NCs displayed bright blue-light emission, with the photoluminescence quantum yield increasing from an initial 6.8% to 69.2%. This study developed an effective strategy to improve the luminescence performance of rotor ligand MOFs, offers a new avenue for the rational design and synthesis of MOFs with superior luminescent properties.

**Supplementary Information:**

The online version contains supplementary material available at 10.1007/s40820-025-01917-8.

## Highlights

- The luminescence performance of MIL-140A is successfully improved via pressure treatment strategy with a significant increase of photoluminescence quantum yield from the initial 6.8% to 69.2%.
- Pressure treatment boosts the host–guest interactions by inducing aperture contraction, which enhances the structural rigidity and thus enables efficient emission.

**Supplementary Information:**

The online version contains supplementary material available at 10.1007/s40820-025-01917-8.

## Graphical abstract

**Figure:**
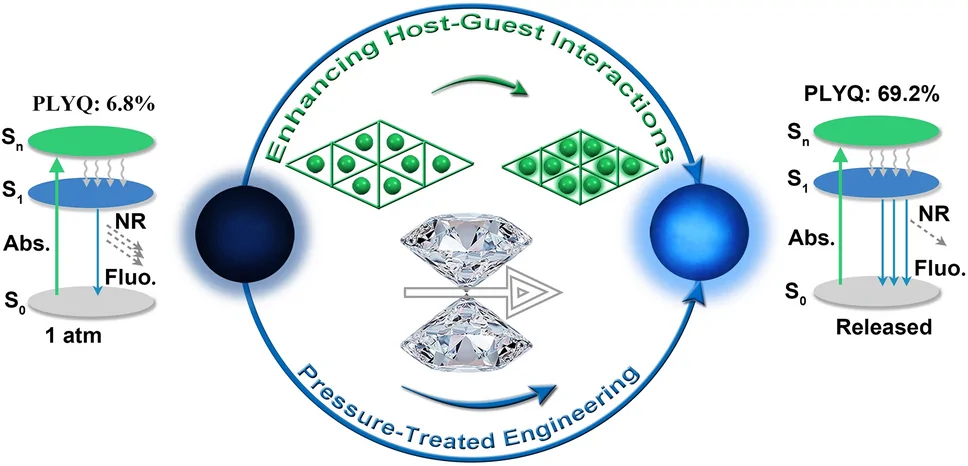

## Introduction

**Fig. 1:**
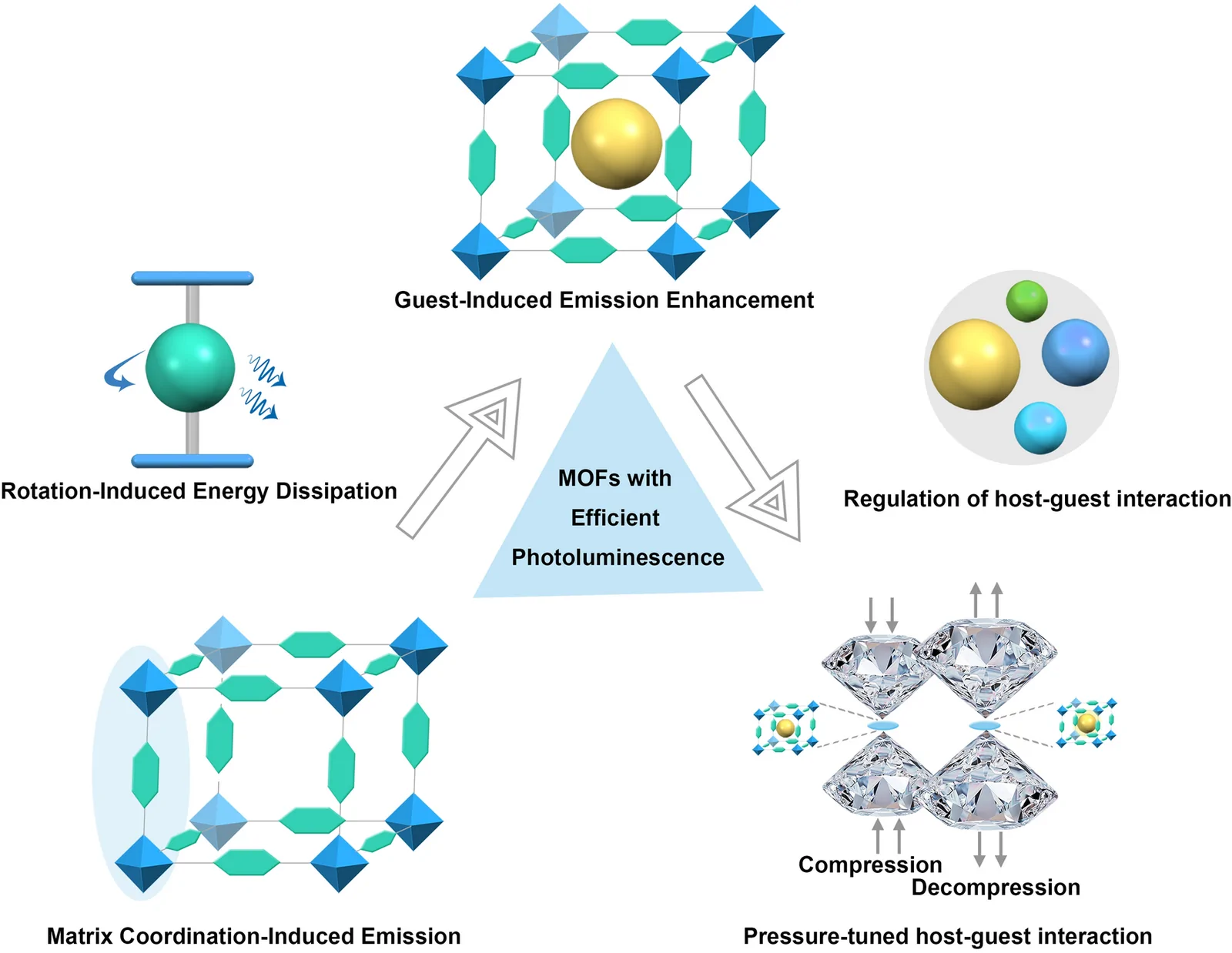
Scheme for the preparation of high-performance LMOFs

Metal–organic frameworks (MOFs) exhibit unique luminescent properties due to their diverse emission centers, rich emission pathways, and rigid coordination environments [1–6]. The structural tunability of MOFs further broadens their potential applications in chemical sensing, biological imaging, optical anti-counterfeiting, LED lighting, and other fields [7–12]. The key to constructing efficient luminescent MOFs (LMOFs) lies in the design of rigid coordination environments. Based on the matrix coordination-induced emission effect, anchoring organic chromophore within the framework structure can effectively suppress non-radiative energy losses caused by chromophore vibrations, thereby enabling the preparation of effective LMOFs [13–15]. Additionally, introducing suitable guest molecules and leveraging host–guest interactions can further stabilize the chromophore conformation, which significantly optimizes the luminescent performance of the materials [16–19]. However, these strategies often require complex structural design and laborious chemical synthesis for regulating chromophore conformation and modulating host–guest interactions (Fig. 1). For MOFs constructed from configurationally simple and widely applicable ligands, the precise modulation of intermolecular interactions within the framework to lock the chromophore conformation and enhance structural rigidity is critical for designing high-performance LMOFs.

Pressure treatment, which serves as a clean and efficient approach to modulate the crystal and electronic structures of materials without altering their chemical composition, has demonstrated remarkable control over the emissive properties of diverse materials [20, 21]. By regulating molecular configurations, intermolecular interactions, and electronic coupling, pressure has induced superior emissive performance in materials including organic molecules, inorganic semiconductors, perovskites, and MOFs [22–25]. More critically, the pressure-induced exceptional emission can be retained under ambient conditions by raising phase transition energy barriers based on steric hindrance and hydrogen-bonding synergies [26–31]. As a prominent class of stimuli-responsive materials, MOFs are rich in easily regulated intermolecular interactions [32–34]. Modulating these interactions via pressure treatment enables the construction of rigid coordination environments and the achievement of efficient emission in MOFs [35–38]. Currently, pressure-modulated hydrogen bonding can effectively enhance the emission of MOFs by increasing the rotational barrier of the rotor and improving the planarization of the chromophore conformation under ambient conditions [27, 28]. However, current research on high-pressure regulation of the PL properties in MOFs has primarily focused on optimizing luminescence by modulating the host framework structure, with little attention paid to guest molecules within the MOF framework [26–31]. Thus, it remains a pressing challenge to further leverage the interactions between guest molecules in the pores and the host framework to develop pressure-responsive efficient emission systems in MOF materials constructed from rotor ligands with highly ordered pore structures.

Here, we achieve pressure-induced emission enhancement in rotor ligand BDC-based MIL-140A nanocrystals (NCs). More importantly, the engineered MIL-140A NCs exhibit bright blue-light emission with photoluminescence quantum yield (PLQY) up to 69.2% from initial 6.8% after the pressure is completely released. In situ high-pressure structural analyses reveal that the pore contraction of MIL-140A NCs leads to the enhancement of interactions between guest molecules and linkers under pressure. The enhanced host–guest interaction, which contributes to the structural rigidity of the framework, effectively limits vibration of the framework and rotation of the rotor, leading to the emission enhancement. After completely releasing the pressure, the engineered host–guest interactions are retained under ambient conditions, which is responsible for the bright blue-light emission of engineered MIL-140A NCs. Our work successfully improves the luminescence efficiency of MIL-140A NCs under ambient conditions, providing a promising strategy for developing effective LMOFs materials.

## Experimental Section

### Preparation of MIL-140A and MIL-140C

The syntheses of MIL-140A and MIL-140C were conducted with reference to previously reported protocols [39]. ZrCl_4_ (Alfa Aesar, 99.5+%), 1,4-benzenedicarboxylic acid (H_2_BDC) (Aldrich, 98%), 4,4'-biphenyl-dicarboxylic acid (H_2_BPDC) (Aldrich, 98%), N, N-Dimethylformamide (DMF), acetic acid (CH_3_CO_2_H), and acetone were used as received without further purification.

MIL-140A series: 2 mmol of H_2_BDC and 1 mmol of ZrCl_4_ were dissolved in 25 mL of DMF. The solution was transferred to a 50 mL Teflon-lined stainless steel autoclave, which was then placed in an oven and heated at 220 °C for 16 h. MIL-140A: The product of hydrothermal reaction was washed three times with DMF and then dried in air. The vacuum-dried MIL-140A: The product of hydrothermal reaction was washed three times with DMF and acetone, followed by dynamic vacuum drying in a vacuum oven, heated at 160 °C for 10 h with continuous vacuum pumping. MIL-140A(acetone): The product of hydrothermal reaction was washed three times with DMF and acetone, then soaked in acetone for 3 days, with fresh acetone replaced every 24 h during this period.

MIL-140C: 2 mmol of H_2_BPDC and 1 mmol of ZrCl_4_ were mixed in 25 mL of DMF and 286 μL (5 mmol) of CH_3_CO_2_H. The mixture was stirred at room temperature for 5 min, then transferred to a 50 mL Teflon-lined stainless steel autoclave. The autoclave was placed in an oven and heated at 220 °C for 12 h. The resulting product was washed three times with DMF and then dried in air.

### High-Pressure Generation

The high pressure was generated by a symmetrical diamond anvil cell (DAC) device with an anvil size of 400 μm. A diamond anvil-sized groove with a thickness of 45 μm was pre-pressed in the center of a T301 stainless steel spacer with a thickness of 250 μm. Then, a 150 μm diameter hole was punched in the center of the groove using a laser punch to serve as the sample cavity. Before the test, a ruby ball was placed in the sample cavity, and pressure calibration was performed by R1 fluorescence spectroscopy of the ruby. All tests did not require additional pressure-holding time before spectroscopic measurement after pressure loading and calibration. The large volume pressure-treated MIL-140A sample was prepared by a Kawai-type high-capacity press.

### In Situ High-pressure Measurements

In situ high-pressure photoluminescence (PL) spectroscopy measurements were performed using a QE65000 fiber optic spectrometer (Ocean Optics). A 355 nm UV DPSS laser was used as the excitation source. PL images of MIL-140A NCs were captured using a Nikon Eclipse TI-U camera mounted on a Canon Eos 5D Mark II microscope.

In situ high-pressure angle-dispersive X-ray diffraction (ADXRD) measurement was performed at the BL15U1 beamline in Shanghai Synchrotron Radiation Facility (SSRF). CeO_2_ was used as the standard sample for calibration. The Bragg diffraction rings were recorded by a Mar-165 CCD detector and integrated into a one-dimensional profile using the FIT2D program.

In situ high-pressure infrared (IR) absorption spectroscopy measurement was carried out at room temperature using a Bruker Vertex 70 V FT-IR spectrometer (Bruker Optik GmbH, Germany) equipped with a nitrogen-cooled mercury–cadmium–telluride (MCT) detector. KBr was used as pressure transfer medium in IR experiments.

In situ high-pressure Raman spectra were recorded using a spectrometer equipped with a liquid-nitrogen-cooled CCD (iHR 550, Symphony II, Horiba Jobin Yvon). A 785 nm single-mode DPSS laser was utilized to excite the sample.

## Results and Discussion

### Crystal Structure, Morphology and Pressure-Modulated Luminescent Properties

**Fig. 2:**
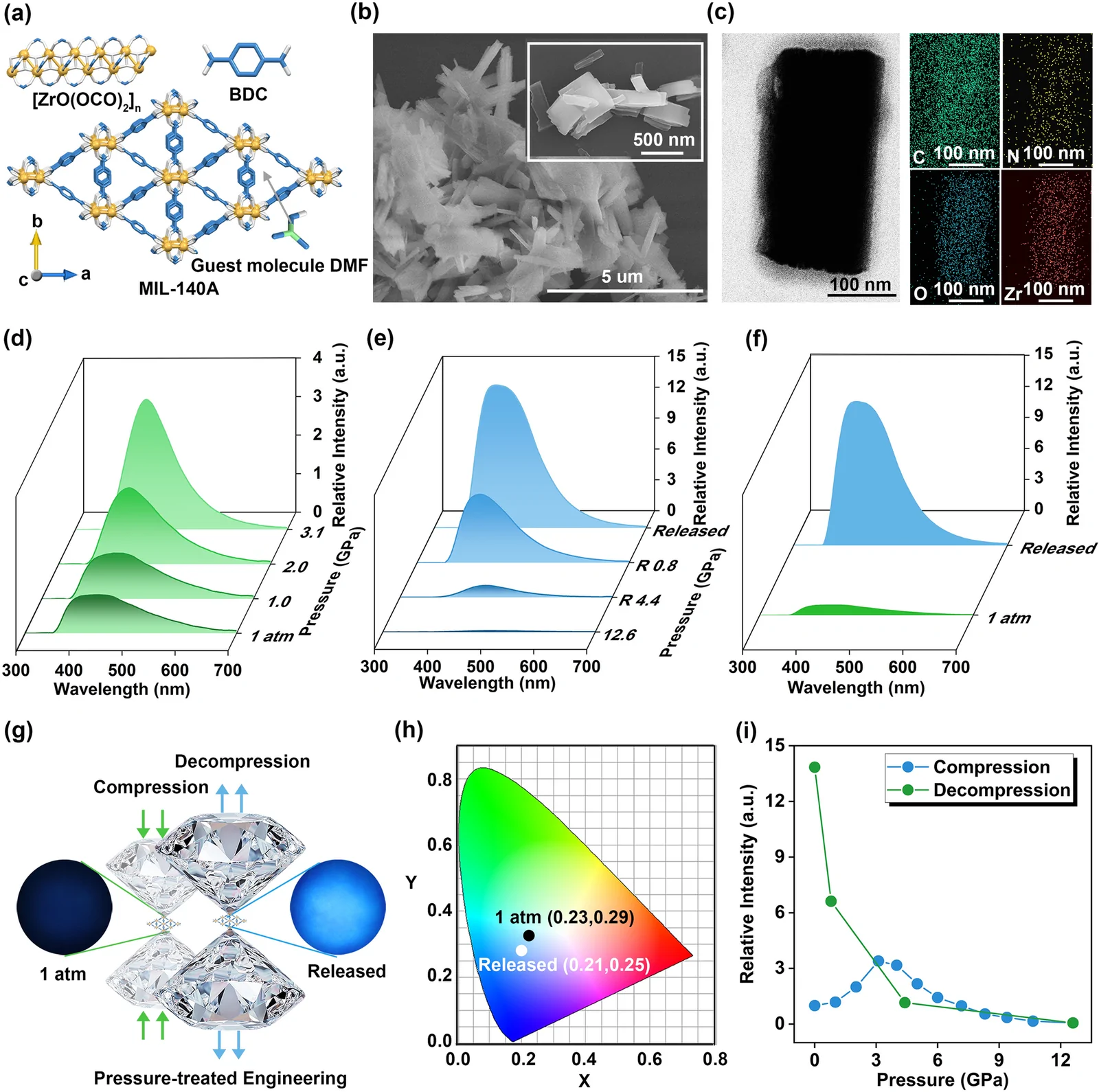
Crystal structure, morphology, and pressure-dependent luminescence properties of MIL-140A NCs. **a** Crystal structure of MIL-140A NCs at ambient conditions (H atoms are omitted for clarity). **b** Scanning electron microscopy image, and **c** energy-dispersive X-ray spectroscopy elemental mapping of MIL-140A NCs under ambient conditions. In situ high-pressure PL spectra of MIL-140A NCs during **d** compression to 3.1 GPa, **e** decompression from 12.6 GPa and **f** before and after pressure treatment. **g** PL photographs and **h** CIE chromaticity of MIL-140A NCs before and after pressure treatment. **i** Evolution of the emission intensity with pressure in MIL-140A NCs

In this study, MIL-140A, namely ZrO(BDC), is synthesized using ZrCl_4_ and H_2_BDC as precursors in DMF solvent at 220 °C [39]. MIL-140A exhibits a monoclinic structure with the space group C2/c under ambient conditions. The inorganic subunit, composed of zirconium oxide chains oriented along the c-axis, is connected to six neighboring zirconium oxide chains via BDC linkers, constructing triangular c-axis-oriented channels (Fig. 2a). MIL-140A features well-defined one-dimensional channels and rigid organic ligands, a structural characteristic that simplifies investigating host–guest interactions under pressure. This renders it uniquely advantageous for exploring the regulatory mechanisms through which host–guest interactions modulate luminescence in rotor-based MOFs. Scanning electron microscopy images reveal that the as-synthesized MIL-140A forms uniformly lamellar nanocrystals (NCs) (Fig. 2b). High-resolution scanning electron microscopy images with well-defined lattice fringes demonstrate the high crystallinity of the MIL-140A NCs. The observed fringe spacing of 0.29 nm corresponds to the (222) crystallographic plane (Fig. S1). Energy-dispersive X-ray spectroscopy elemental mapping reveals a homogeneous spatial distribution of O, C, N, and Zr elements throughout the MIL-140A NCs. The observed N elements clearly confirm the presence of DMF guest molecules in the apertures, since the N elements are exclusively derived from solvent molecules (Fig. 2c). Notably, BDC is a typical rotator and its rotational motion within the framework leads to excited-state energy dissipation. As a result, MIL-140A NCs exhibit poor luminescence performance with PLQY of only 6.8% under ambient conditions (Fig. S2).

Capitalizing on the inherent structural tunability of MOFs, modulation of the intermolecular interactions via pressure to achieve the fixation of chromophore conformation has become a promising strategy for improving luminescence performance. To explore the effect of pressure on the emission properties of MIL-140A NCs, we conduct in situ high-pressure PL measurements. As shown in Fig. 2d, MIL-140A NCs exhibit pressure-induced blue-light emission enhancement up to 3.1 GPa. At higher pressures, the PL intensity gradually decreases, with complete emission quenching occurring at 12.6 GPa (Fig. S3). Notably, a remarkable enhancement of the emission intensity of MIL-140A NCs is observed during decompression (Fig. 2e). After the pressure is completely released, a bright blue emission is obtained with the Commission Internationale de l’Eclairage (CIE) chromaticity coordinates of (0.21, 0.25) and can be maintained under ambient conditions over 3 months (Figs. 2f-h and S3). The evolution behavior of the emission intensity of MIL-140A NCs with pressure is shown in Fig. 2i, directly demonstrating the fascinating stimulus-responsive PL behavior of MIL-140A NCs. The luminescence performance of MIL-140A NCs is successfully optimized by pressure treatment to achieve the bright blue emission under ambient conditions. Furthermore, to accurately clarify the emission mechanism of MIL-140A, density of states and natural transition orbital analyses are performed (Fig. S4). The results show that the energy bands of MIL-140A are primarily contributed by C atoms and O atoms. Additionally, natural transition orbital analyses reveal that the highest occupied molecular orbital (HOMO) and the lowest unoccupied molecular orbital (LUMO) of MIL-140A are localized on the organic BDC ligands. This suggests that MIL-140A is based on ligand emission.

### Structural Evolution with Pressure

**Fig. 3:**
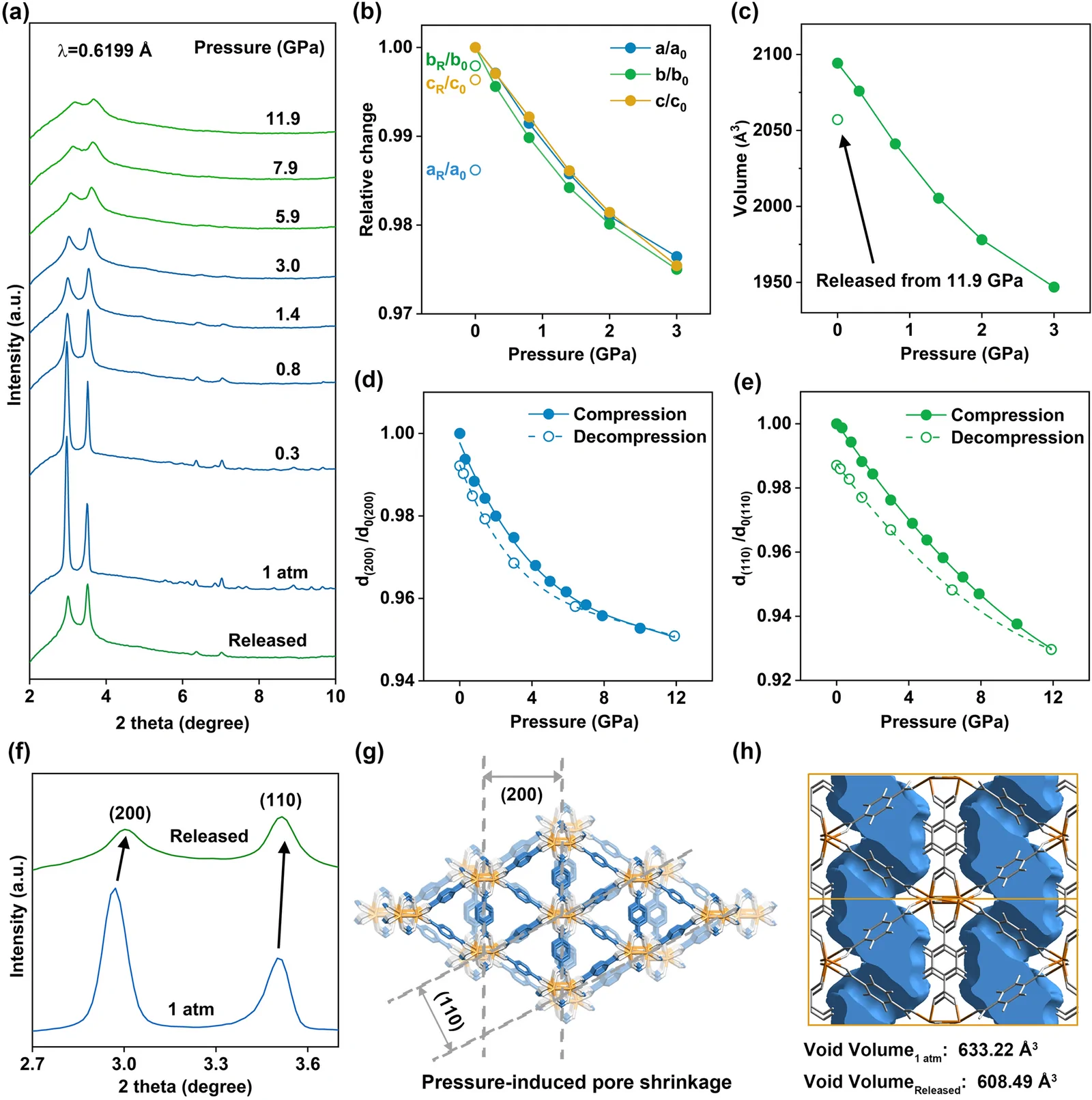
Evolution of crystal structure with pressure in MIL-140A NCs. **a** In situ high-pressure ADXRD patterns of MIL-140A NCs. **b** Compression rate of lattice constants at different pressures in MIL-140A NCs. **c** Evolution of unit cell volume with pressure in MIL-140A NCs. The compression rate of **d** *d*_(200)_ and **e** *d*_(110)_ spacings as a function of pressure. **f** Amplified ADXRD pattern of MIL-140A NCs before and after pressure treatment in the range of (200) and (110) crystal planes. **g** Schematic diagram of (200) and (110) crystal planes. **h** Change in void volume before and after pressure treatment (The voids of two unit cells are shown for better display of the void structure)

The modulation of luminescence properties is closely correlated with structural evolution under pressure. In order to elucidate the intrinsic mechanism of pressure modulation on luminescence properties of MIL-140A NCs, we perform in situ high-pressure structural analyses. In situ high-pressure ADXRD spectra show that all Bragg diffraction peaks gradually shift to higher angles with the increase of pressure, accompanied by the broadening and disappearance of partial peaks (Fig. 3a). To obtain more in-depth information about structural transformations under pressure, we perform Rietveld refinement of the ADXRD patterns (Fig. 3b, c). The results show that up to 3.0 GPa, MIL-140A NCs maintain the C2/c phase, in which the a, b, and c axes exhibit similar compression rates, exhibiting normal lattice contraction (Fig. S5 and Table S1). Above 3.0 GPa, most diffraction peaks vanish, with the exception of those associated with the (200) and (110) crystal planes, indicating a decrease in the crystallinity of MIL-140A NCs. In this regard, we are unable to perform a precise Rietveld refinement, yet the variation of (200) and (110) crystal spacing with pressure during pressure cycling at 11.9 GPa is documented in Fig. 3d, e. The (200) and (110) crystal spacing are consistently shortened during compression, and enlarged during decompression. Notably, the compressed crystal faces are not fully recovered and remain compressed after the pressure is fully released, which is confirmed by the enlarged local diffraction peaks in the ADXRD patterns before and after pressure treatment of 11.9 GPa (Figs. 3f and S6). The decrease in cell parameters and cell volume after the pressure treatment is consistent with the compression of the (200) and (110) planes. The compression of the (200) and (110) crystal planes results in the narrowing of the triangular channels, as further evidenced by the pore volume of the unit cells before and after the pressure treatment (Figs. 3g and h). The shrinkage of the voids significantly modulates the interactions between the DMF molecules within the channels and the framework.

### Mechanisms of Pressure-regulated Luminescence Performance

**Fig. 4:**
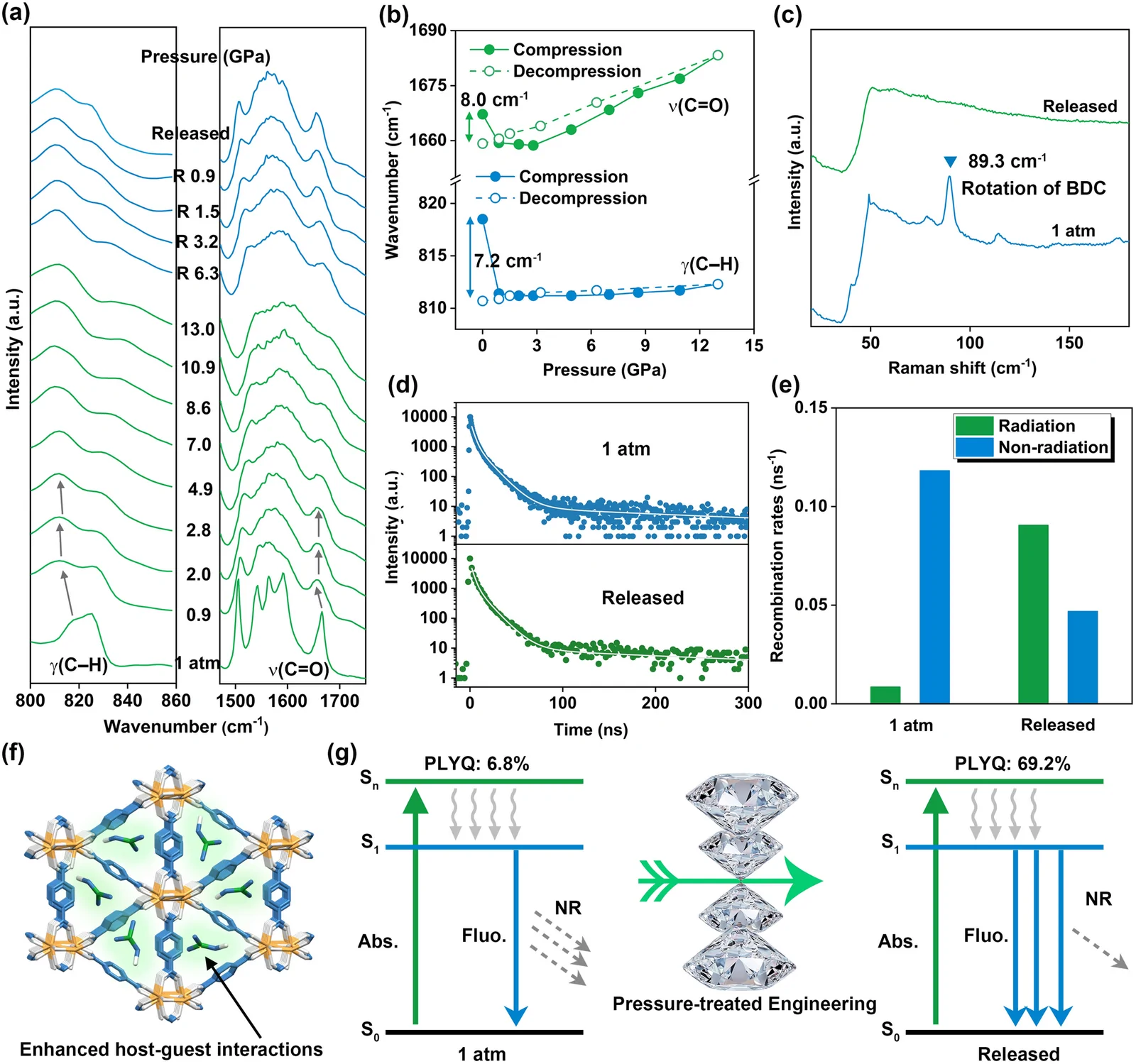
Mechanisms of pressure-regulated luminescence performance in MIL-140A NCs. **a** Amplified in situ high-pressure IR spectra in the region of γ(C−H) and ν(C=O). **b** Wavenumber γ(C−H) and ν(C=O) as a function of pressure. **c** Amplified Raman spectra before and after pressure treatment in the region of rotation of BDC. **d** PL decay curves and **e** radiative and non-radiative recombination rates before and after pressure treatment of 12.0 GPa. **f** Schematic illustration of the pressure-regulated host*–*guest interactions. **g** Schematic diagram of the PL mechanism in MIL-140A NCs before and after pressure treatment. (S_0_, S_1_ and S_n_ represent the ground singlet state, the first excited singlet state and higher excited singlet state, respectively; Abs.: absorption; Fluo.: fluorescence; NR: non-radiative)

To further reveal the changes in intermolecular interactions under pressure, we perform in situ high-pressure IR absorption spectroscopy on MIL-140A NCs (Fig. S7 and Table S2). Typically, under pressure, the interatomic distance decreases and the chemical bonding is shortened, which is manifested as a blue shift of the IR vibrational peaks. Most of the IR absorption peaks exhibit normal blue-shifting in MIL-140A NCs (Fig. 4a, b). However, the IR peaks at 811.3 and 1659.2 cm^−1^, attributed to the out-of-plane bending vibration γ(C−H) of aromatic C−H and the stretching vibration ν(C=O) of DMF molecule, respectively, are shifted to a lower wavenumber below 2.8 GPa, which suggests that the relevant vibrations are limited under pressure [40, 41]. The associated vibrational restriction can be attributed to enhanced C–H···O–C hydrogen bonding interactions between the ligand and the guest molecules. The restriction of vibrations can effectively suppress non-radiative transition processes, which accounts for the enhanced emission of MIL-140A NCs under 3.1 GPa. At higher pressures, the relevant vibration peaks continue to blue shift, indicating that the enhanced vibration effect caused by pressure-induced pore size contraction exceeds the vibration restriction caused by host*–*guest interactions. In addition, the in situ high-pressure Raman scattering spectra revealed a continuous blue shift in Raman vibrational peaks of MIL-140A NCs, accompanied by peak broadening, attenuation, and even the disappearance of some peaks under pressure (Fig. S8). This observation aligns with the ADXRD analysis, indicating that pressure induces lattice contraction and progressive amorphization in MIL-140A NCs. The pressure-induced structural distortions and ligand bending disrupt the conjugated structure of chromophores, which accounts for the PL weakening in MIL-140A NCs when the pressure exceeds 3.1 GPa. Notably, after the pressure is fully released from 13.0 GPa, γ(C−H) and ν(C=O) exhibit redshifts of 7.2 and 8.0 cm^−1^ compared to those before pressure treatment, respectively, indicating that the vibration peaks remain confined. Upon complete pressure release, the BDC rotational peak at 89.3 cm^−1^ vanishes, suggesting that the rotation of the BDC ligand is restricted (Fig. 4c) [42]. The restriction of vibration and rotation results in efficient emission of MIL-140A after pressure treatment.

To qualitatively characterize the transition process, the recombination rates have been calculated based on the time-resolved photoluminescence spectroscopy before and after the pressure treatment (Fig. 4d and e). After pressure treatment, radiative recombination rate (k_r_) increases to 0.0952 ns^−1^ from the initial 0.0086 ns^−1^ and non-radiative recombination rate (k_nr_) decreases to 0.0424 ns^−1^ from the initial 0.1183 ns^−1^ in MIL-140A NCs. Combined with in situ high-pressure structural analysis, the pressure-induced contraction of pore apertures enhances the interaction between guest DMF molecules and the BDC ligands within the framework (Fig. 4f). The strengthened host*–*guest interactions effectively increase the framework rigidity and constrain the vibration and rotation of frameworks, which effectively reduces the non-radiative transition energy loss (Fig. 4g). As a result, MIL-140A NCs exhibit bright blue emission after pressure treatment with PLQY increasing to 69.2% from the initial 6.8% (Table S3 and Fig. S9). Given the significant impact of guest molecules on the luminescent properties of the material, we further subject MIL-140A to vacuum drying to remove the guest molecule DMF [43]. Thermogravimetric analysis confirms that vacuum drying effectively removes the guest molecule DMF (Fig. S10). Notably, the vacuum-dried MIL-140A NCs exhibit pressure-induced emission reduction, and the emission intensity of engineered MIL-140A NCs after pressure treatment of 12.0 GPa is slightly lower than that before pressure treatment (Fig. S11). In situ high-pressure IR absorption spectroscopy shows that the IR absorption peaks of vacuum-dried MIL-140A exhibit continuous blue shifts and broadening under pressure (Fig. S12 and Table S4). This indicates that pressure induces lattice distortion, which in turn causes ligand bending in vacuum-dried MIL-140A. The bending of the conjugated structure of the chromophore ligands is responsible for the decrease in PL intensity. This confirms that pressure-induced host*–*guest interactions are the key to the enhanced emission of MIL-140A. In addition, we replace DMF with acetone to prepare MIL-140A(acetone). The regulatory effect of pressure on the PL properties of MIL-140A(acetone) is less pronounced than that on MIL-140A, which indicates that the size matching between guest molecules and MOF also influences the regulation of MOFs' PL properties (Fig. S13).

Interestingly, MIL-140C NCs, namely ZrO(BPDC), which share the same ZrO chains, c-axis parameter and topological structure with MIL-140A NCs, exhibit pressure-induced emission reduction until the emission quenches at 12.9 GPa (Figs. S14 and S15). When the pressure is fully released, the emission intensity is lower than that of the initial state in MIL-140C NCs. To carefully analyze the very different emission response behaviors of MIL-140A NCs and MIL-140C NCs to pressure, the detailed in situ high-pressure structural analysis of MIL-140C NCs is performed. In situ high-pressure IR absorption spectra show that all IR peaks in MIL-140C NCs undergo a sustained blue shift under pressure and do not show the vibrational confinement observed in MIL-140A NCs (Fig. S16 and Table S5). In addition, the in situ high-pressure Raman spectra show that the Raman peaks of MIL-140C NCs also exhibit constant blue shift under pressure (Fig. S17). This indicates that the lattice contraction leads to the enhancement of the associated chemical bonding vibrations in MIL-140C NCs. Notably, the pressure-induced blue shift of the C−C stretching vibration *ν*(C−C) between the biphenyl rings of the BPDC ligands is not reversible during decompression. After pressure treatment, C−C is under compression relative to that before pressure treatment [44, 45]. These observations suggest that MIL-140C NCs undergo pressure-induced ligand bending, which is very common in long-chain ligand-based MOFs materials. The bending of the ligand and the framework deformation induced by the ligand bending restrict the radiative transitions of the ligand chromophore, which is the reason for the pressure-induced emission diminution in MIL-140C NCs.

Although MIL-140A NCs and MIL-140C NCs share similar structures, they exhibit entirely different luminescence responses to pressure, with MIL-140A NCs showing pressure-induced emission enhancement and MIL-140C NCs displaying emission quenching. This stark contrast in pressure-responsive luminescence behavior is primarily attributed to their distinct pore sizes. MIL-140A NCs possess a smaller pore size of about 3.2 Å, where applied pressure induces pore contraction, thereby strengthening host*–*guest interactions. The enhanced hydrogen bonding between the DMF guest molecules and the BDC ligands restricts framework vibrations and ligand rotations under pressure, ultimately improving the emission efficiency of MIL-140A NCs. In contrast, MIL-140C NCs feature a larger pore size of about 5.7 Å. In this case, the distance between guest molecules and ligands is relatively large, and lattice contraction under pressure is insufficient to enable strong host*–*guest interactions with the similarly sized DMF molecules. Instead, pressure more readily induces bending of the elongated ligands, thereby restricting the radiative transitions of the chromophoric ligands and leading to reduced emission efficiency in MIL-140C NCs.

## Conclusions

In conclusion, this study successfully achieves efficient blue-light emission in rotor ligand-based MOFs through pressure treatment strategy. Pressure treatment induces the contraction of one-dimensional channels in the MIL-140A NCs, which significantly enhances the interaction between DMF molecules within the channels and the ligands of the main framework. The enhanced host*–*guest interaction can effectively inhibit the vibration of the guest molecules and the rotation of the ligands, thereby reinforcing the framework rigidity. This structural rigidity greatly reduces the non-radiative transition energy loss and ultimately achieves efficient emission under ambient conditions. Interestingly, the structurally analogous MIL-140C NCs exhibit markedly distinct pressure-response behavior. The expanded pore dimensions in MIL-140C NCs hinder the pressure-mediated enhancement of host*–*guest interactions. Instead, the applied pressure leads to bending of the extended ligands, which causes distortion of the chromophore configuration and consequently diminishes luminescence efficiency. These findings not only elucidate the fundamental structure–emission relationship governing framework flexibility and host*–*guest interactions under pressure, but also provide new insights for the development of highly emissive rotor-based MOF materials.

## Supplementary Information

Below is the link to the electronic supplementary material.

Supplementary file1 (DOCX 6861 KB)
