# Supplementary material for: Pressure-Modulated Host–Guest Interactions Boost Effective Blue-Light Emission of MIL-140A Nanocrystals
Source: Nanomicro Lett. 2025 Sep 15;18:70. doi: 10.1007/s40820-025-01917-8 (PMC12436260; doi:10.1007/s40820-025-01917-8)
Supplement: Supplementary file 1 — Supplementary file1 (DOCX 6861 KB) [file 40820_2025_1917_MOESM1_ESM.docx]

Supporting Information for

Pressure-Modulated Host-Guest Interactions Boost Effective Blue-Light Emission of MIL-140A Nanocrystals

Ting Zhang^1^, Jiaju Liang^1^, Ruidong Qiao^1^, Binhao Yang^1^, Kaiyan Yuan^1^, Yixuan Wang^1*^, Chuang Liu^1*^, Zhaodong Liu^1*^, Xinyi Yang^1*^, and Bo Zou^1^

^1^Synergetic Extreme Condition High-Pressure Science Center, State Key Laboratory of High Pressure and Superhard Materials, College of Physics, Jilin University, Changchun, 130012, China

^*^Corresponding author. E-mail: wangyixuan19@jlu.edu.cn, liuchuang20@jlu.edu.cn, liu_zhaodong@jlu.edu.cn, yangxinyi@jlu.edu.cn

CONTENTS

1. Experimental Procedures S3

2. Supplemental Figures S5

3. Supplemental Tables S22

4. Supplemental References S27

S1. Experimental Procedures

S1.5 Data analyses

Void volume calculations: The structure of MIL-140A NCs was determined by synchrotron X-ray crystallographic analysis. The Crystal Explorer 17.5 program was used to visualize and analyze the void volume of MIL-140A NCs before and after pressure treatment [S1].

The analyses of chromaticity coordinates: The chromaticity coordinates (x, y) of MIL-140A NCs before and after pressure treatment were calculated from the spectra data (380–700 nm) using the CIE1931xy.V.1.6.0.2a software package.

Details of Photoluminescence quantum yield (PLQY) calculation: Using the PLQY at ambient pressure as a reference, the PLQY after pressure treatment was calculated by the following formula:

$$\text{ Φ = }\text{Φ}_{\text{0}}\frac{\int\text{F}}{\int\text{F}_{\text{0}}}\text{∙}\frac{\text{Α}_{\text{0}}}{\text{Α}}\text{∙}\frac{\text{n}^{\text{2}}}{{\text{n}_{\text{0}}}^{\text{2}}}\text{ (1)}$$

In which the subscript 0 indicates the reference data at ambient pressure [S2]. Φ is the PLQY, ∫F is the integrated intensity of PL, A is the absorbance intensity at the excitation wavelength 355nm, n is the refractive index. n_0_ is 1.44, which is referenced from a similar MOF material [S3]. Using n_0_ as a reference, n was calculated based on the Clausius-Mossotti equation and Lorentz-Lorenz equation.

$$\text{ }\frac{\text{n}^{\text{2}}\mathbf{-}\text{1}}{\text{n}^{\text{2}}\text{ + 2}}\text{∙}\frac{\text{1}}{\text{ρ}}\text{ = }\frac{\text{4}\text{π}}{\text{3}}\text{∙}\text{N}_{\text{A}}\text{∙}\text{α}\text{ = }\text{R}_{\text{LL}}\text{ (2)}$$

in which the density *ρ* can be calculated from the cell volume. The Lorentz-Lorenz constant R_LL_ is related to polarizability *α*, and these two parameters are unchanged before and after pressure treatment for the same material.

The analyses of PL decay curves: the PL decay curves before and after pressure treatment were fitted by single exponential function:

$$\text{ I(t) = }\mathbf{I}_{\mathbf{0}}\text{ + A*exp(-t/}\text{τ}\text{) }\text{ }\text{ (4)}$$

The analyses of recombination rates: *k*_nr_ and *k*_r_ were calculated by the following equations:

PLQY =*k*_r_/(*k*_r_+*k*_nr_) (7)

*τ* = 1/(*k*_r_+*k*_nr_) (8)

In which the *τ* was obtained from the fitting of PL decay curves.

S2 Supplemental Figures


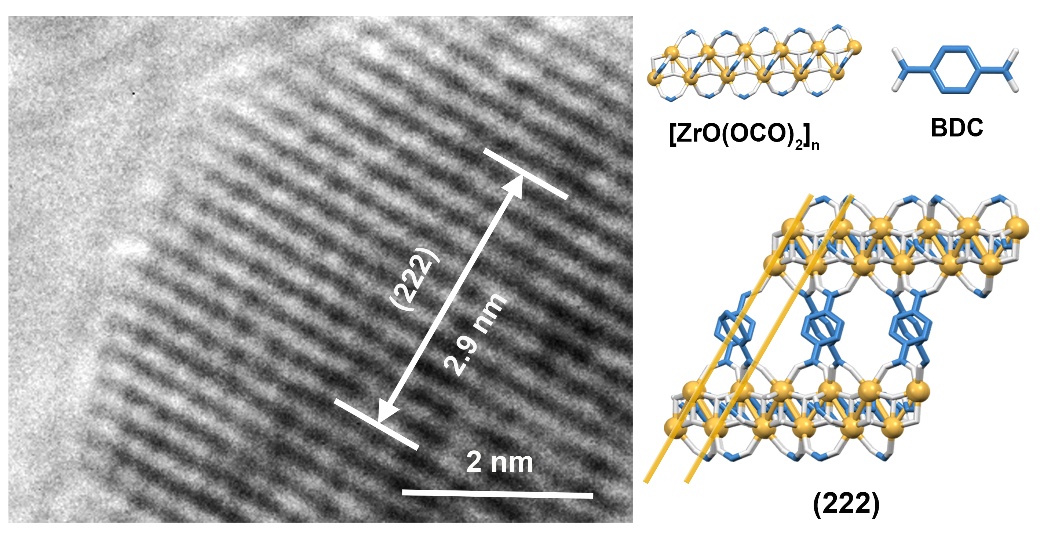


Fig. S1 High-resolution scanning electron microscopy image of MIL-140A NCs under ambient conditions.


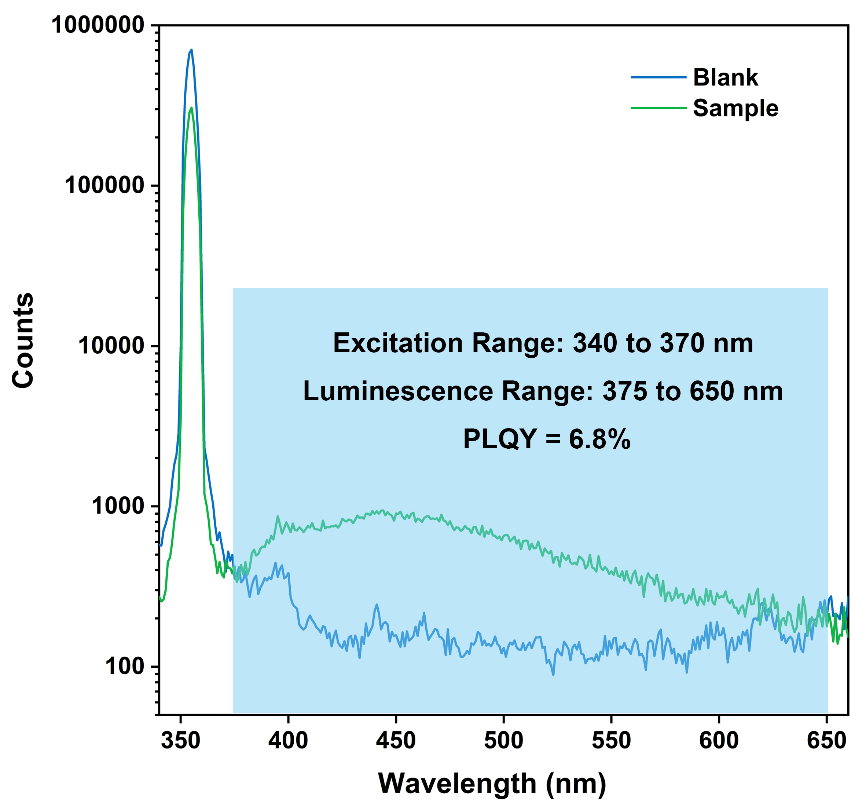


Fig. S2 The absolute PLQY of MIL-140A NCs under ambient conditions measured by an integrating sphere apparatus.


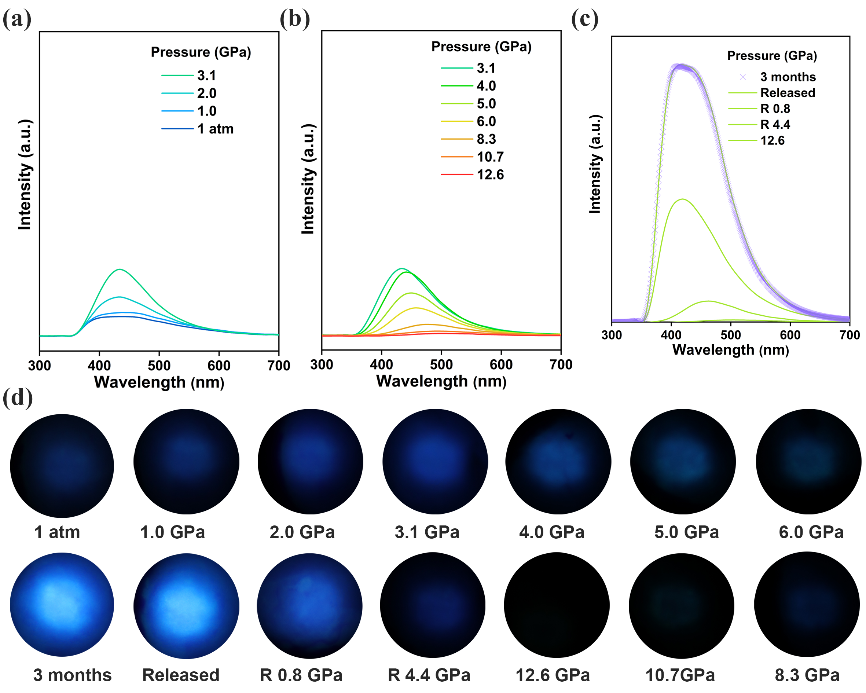


Fig. S3 (a-c) *In situ* high-pressure PL spectra and (d) corresponding PL photographs of MIL-140A NCs under the excitation of 355 nm laser. 3 months means that the pressure-treated sample was remained under ambient conditions for three months.


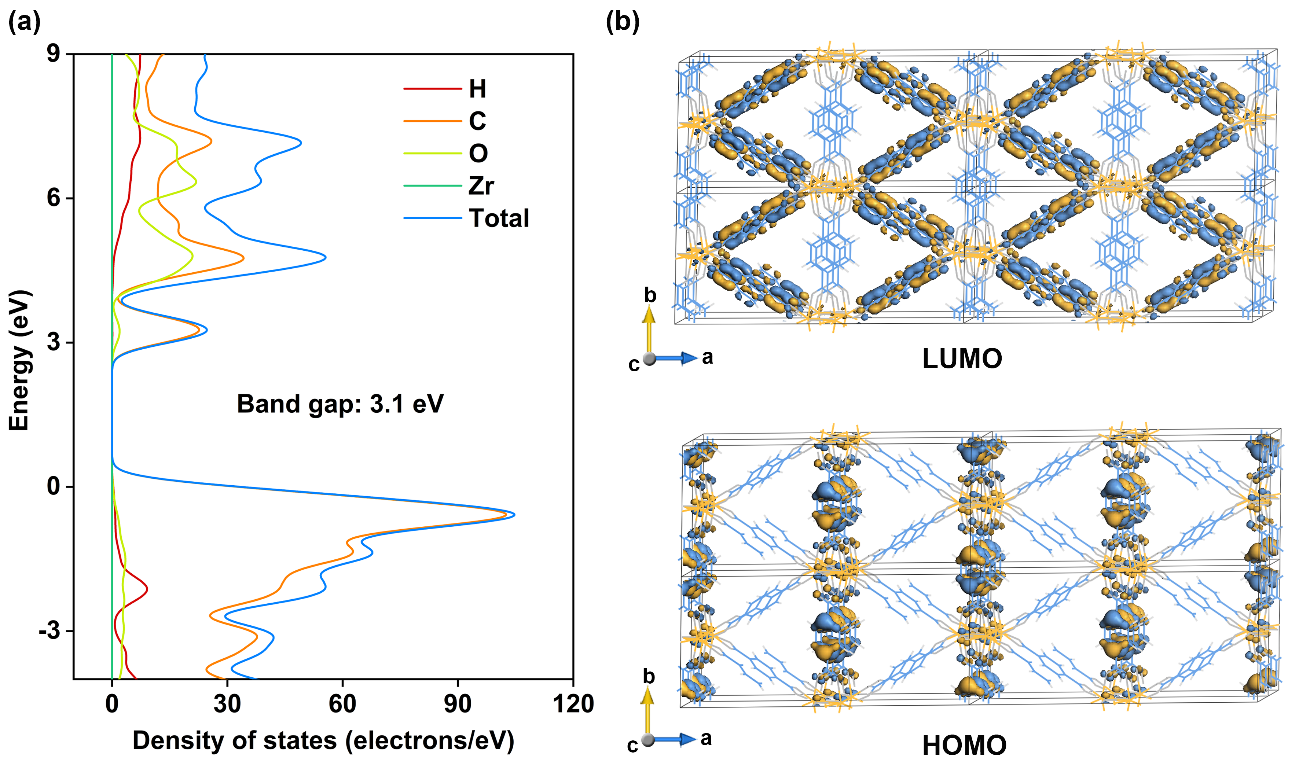


Fig. S4 The (a) density of states and (b) natural transition orbital analyses of MIL-140A NCs under ambient conditions.





Fig. S5 Rietveld refinements of the ADXRD patterns for MIL-140A NCs before and after pressure treatment. Black bars indicate the refined Bragg peak positions and green line represents the difference between observed (red circles) and simulated calculated (blue solid line) diffraction profiles.





Fig. S6 *In situ* high-pressure ADXRD patterns for MIL-140A NCs during decompression.


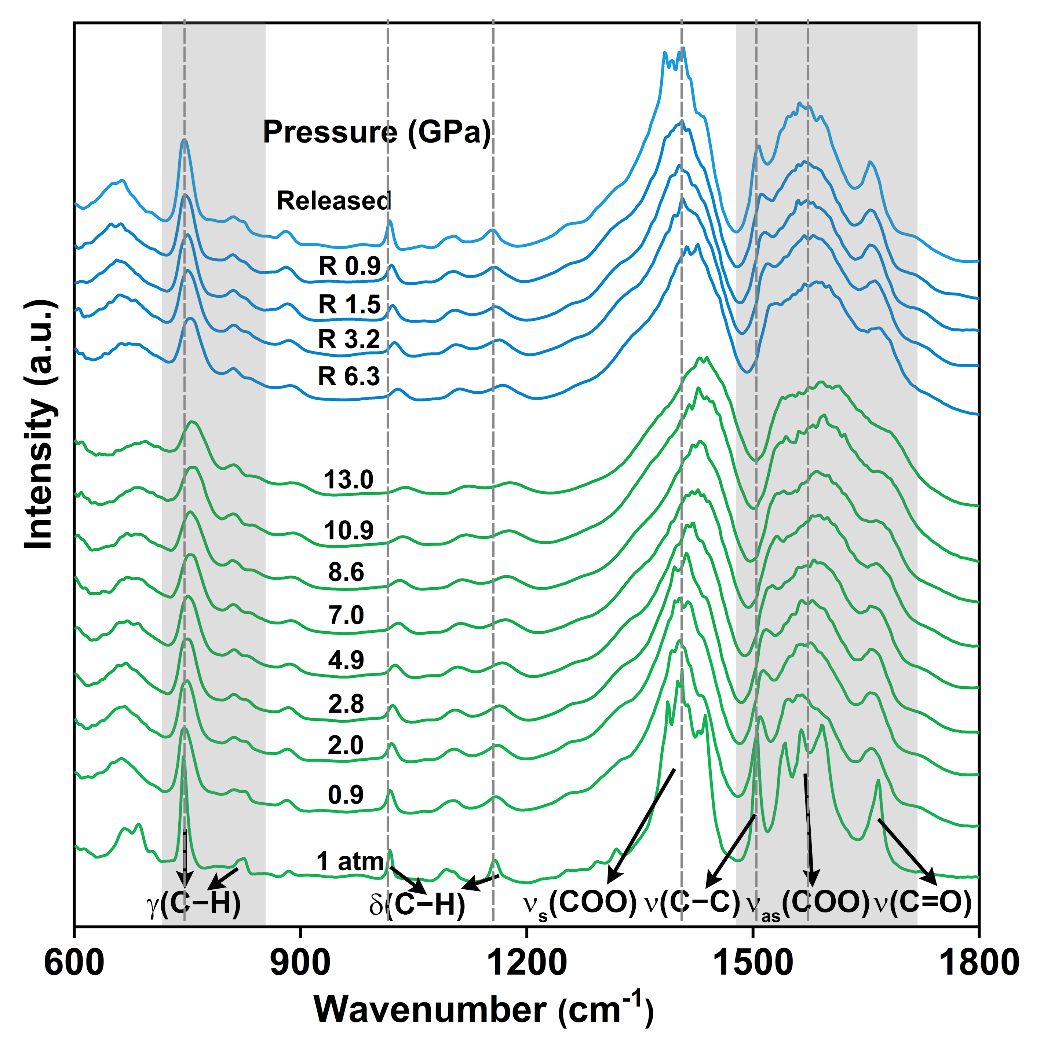


Fig. S7 *In situ* high-pressure infrared (IR) absorption spectra for MIL-140A NCs in the region of 600-1800 cm^-1^. The regions marked in grey correspond to the out-of-plane deformation vibration of aromatic C–H, γ(C–H), and stretching vibration of C=O in DMF molecule, ν(C=O), respectively. Except for peaks of γ(C–H) and ν(C=O), all other IR absorption peaks shift to higher wavenumbers under pressure, accompanied by peak broadening.





Fig. S8 *In situ* high-pressure Raman scattering spectra for MIL-140A NCs in the region of 20-1700 cm^-1^.


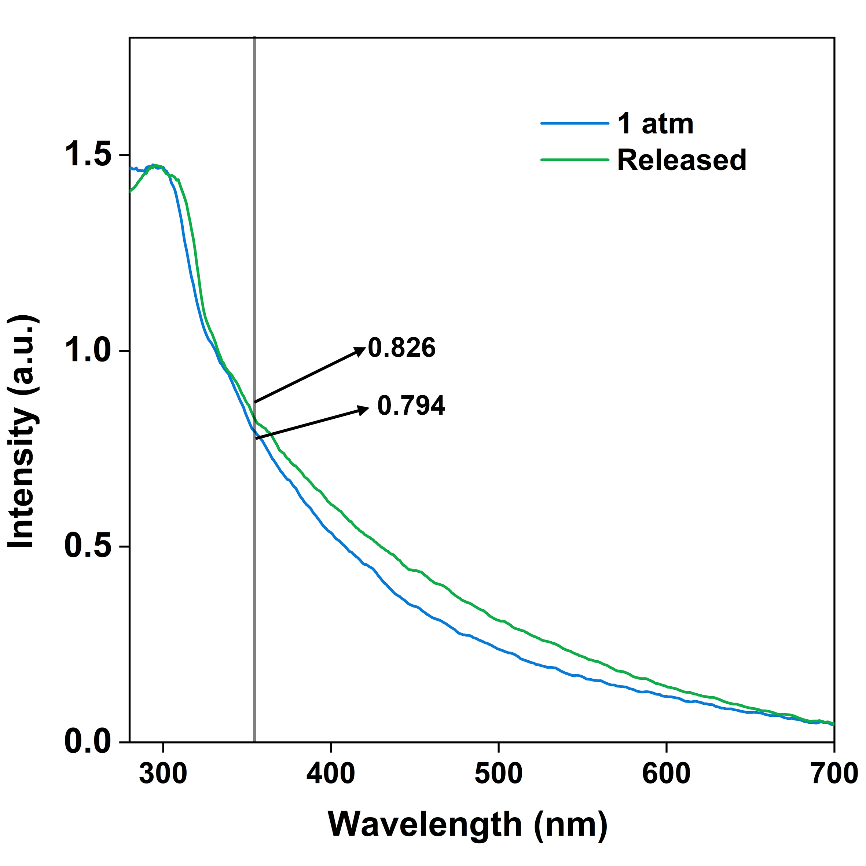


Fig. S9 UV-Vis absorption spectra of MIL-140A NC before and after pressure treatment.


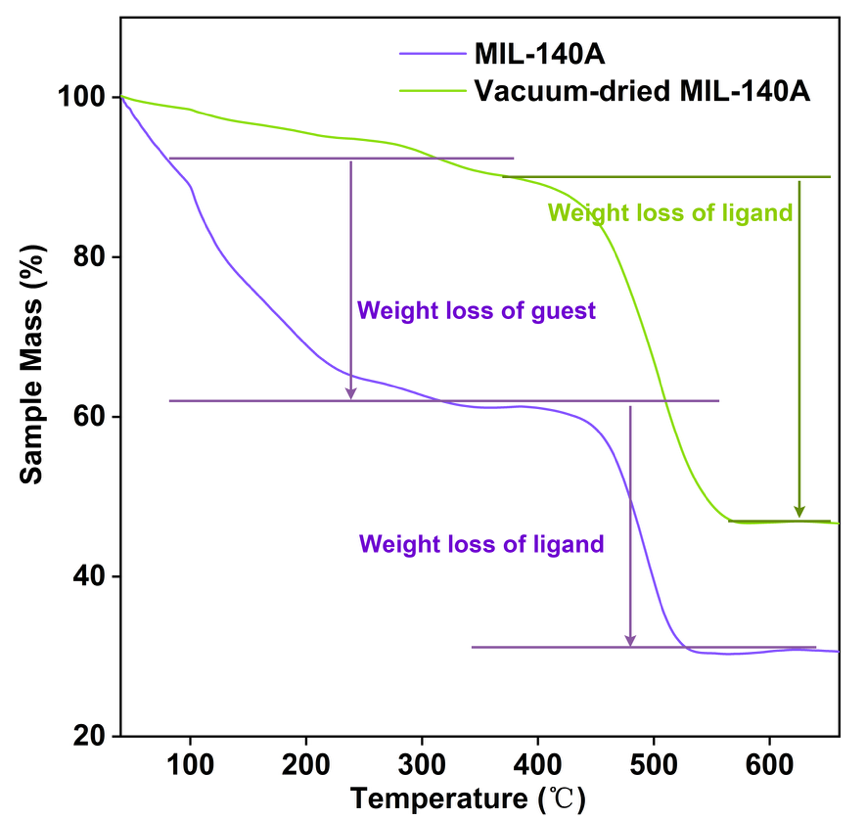


Fig. S10 The thermogravimetric analysis of MIL-140A NC and vacuum-dried MIL-140A NC.


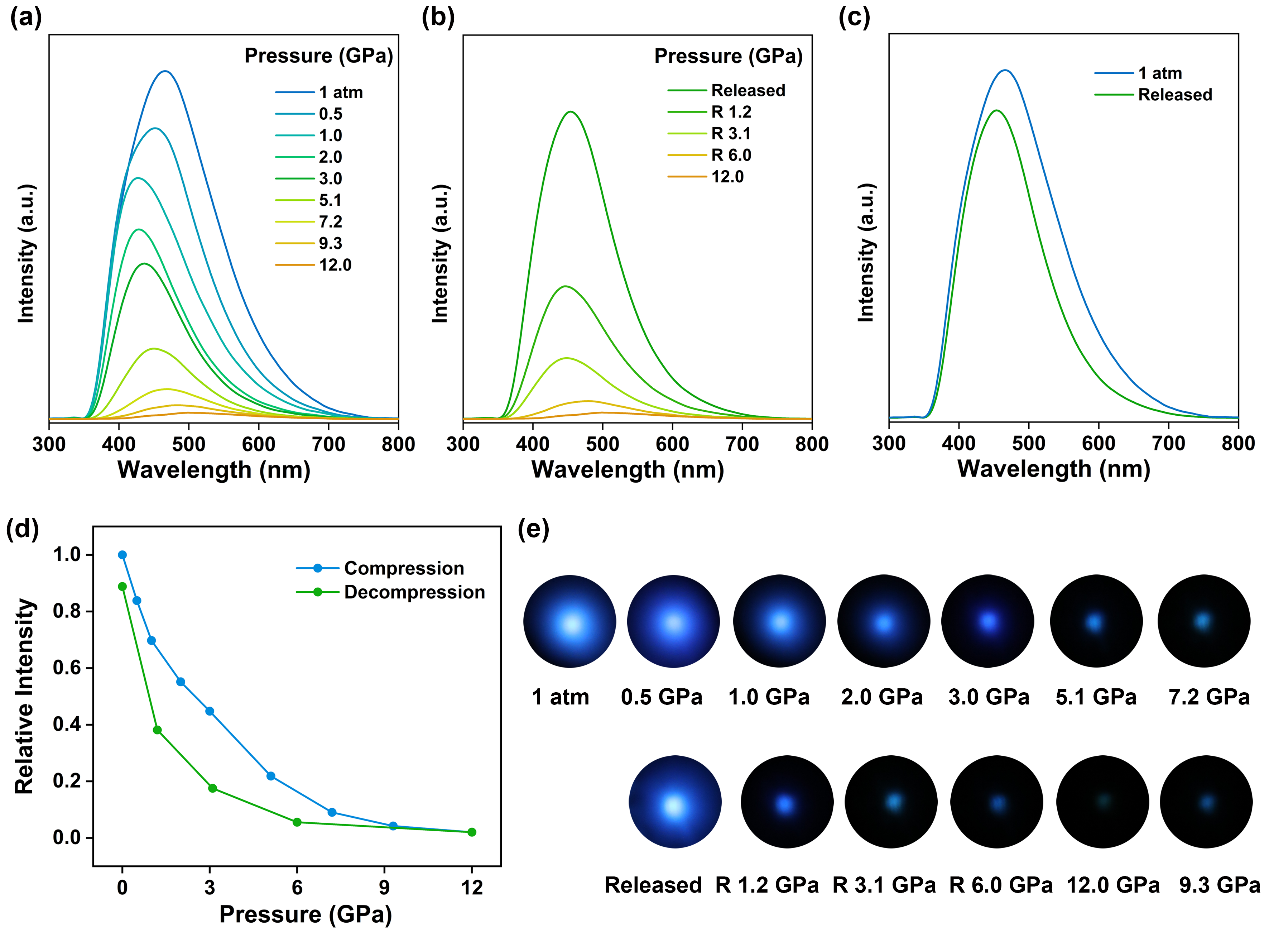


Fig. S11 (a-c) *In situ* high-pressure PL spectra of vacuum-dried MIL-140A NCs under 355 nm laser excitation. (d) Evolution of emission intensity with pressure. (e) Corresponding PL photographs at different pressures.


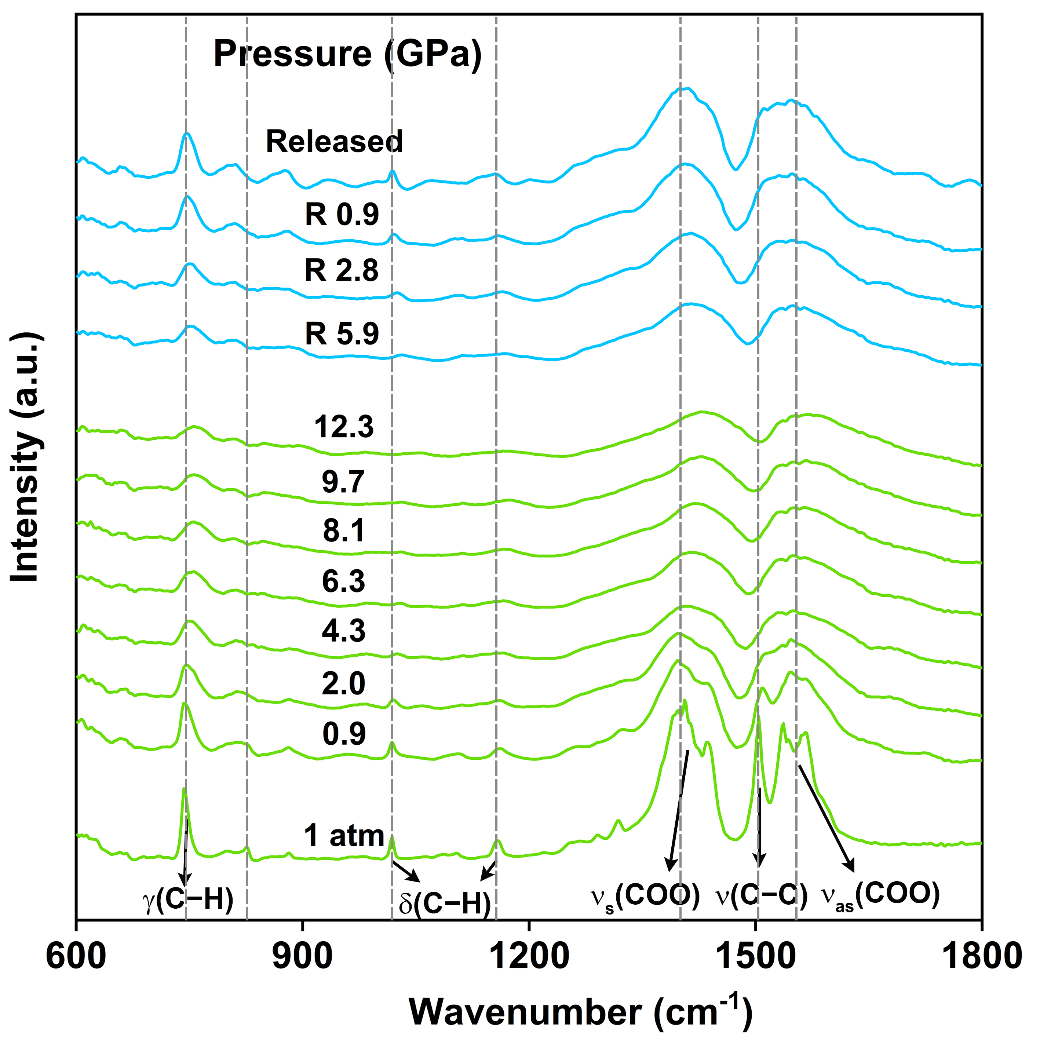


Fig. S12 *In situ* high-pressure IR spectra of vacuum-dried MIL-140A NCs in the region of 600-1800 cm^-1^. All IR absorption peaks shift to higher wavenumbers under pressure, accompanied by peak broadening. This indicates that vacuum-dried MIL-140A undergoes structural contraction and a certain degree of structural distortion under pressure.


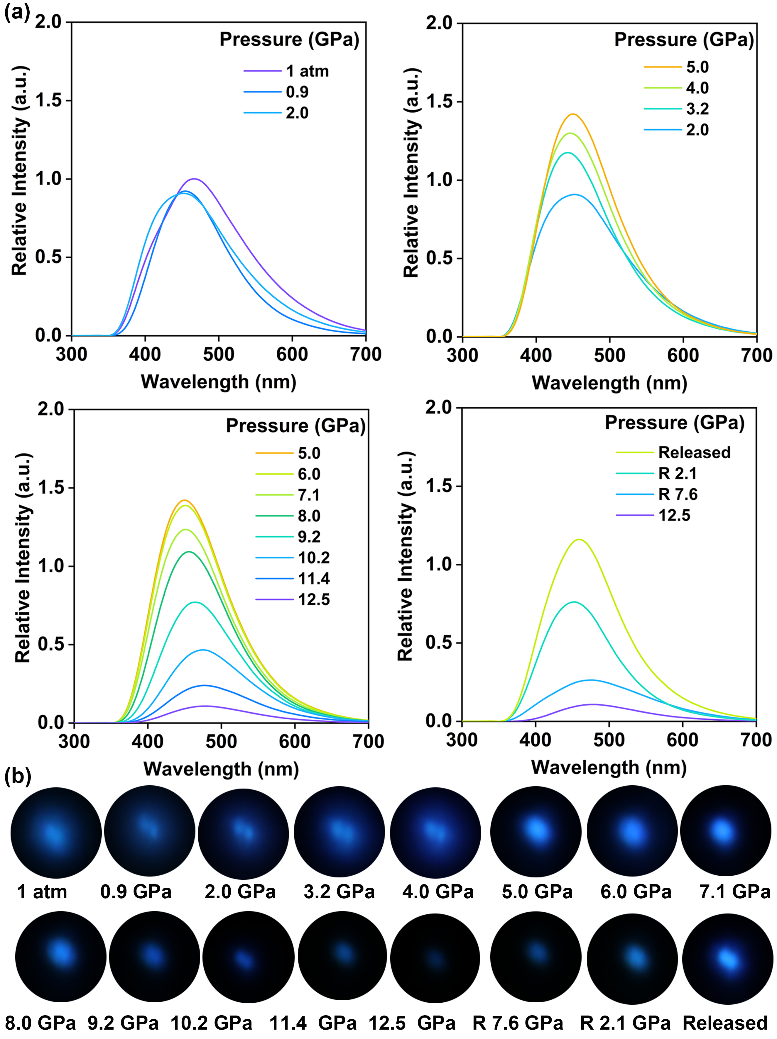


Fig. S13. (a) *In situ* high-pressure PL spectra of MIL-140A(acetone) NCs under 355 nm laser excitation. (b) Corresponding PL photographs at different pressures. The as-synthesized MIL-140A(acetone) exhibits a PL intensity trend of first decreasing, then increasing, and finally decreasing under pressure. After the pressure is completely released from 12.3 GPa, the PL intensity of MIL-140A(acetone) is slightly higher than that before pressure treatment. Compared with MIL-140A, the pressure range for pressure-induced fluorescence enhancement increases from 0–3.1 GPa to 2.0–5.0 GPa, and the magnitude of pressure-induced luminescence enhancement decreases significantly. Notably, for smaller guest molecules, a higher pressure is required to shrink the pore size of MOFs to a sufficiently small dimension, thereby enhancing the host-guest interactions. Furthermore, after complete release of pressure, the enhancement effect of host-guest interactions is poorer for guest molecules that are much smaller than pore size. This may account for the suboptimal regulation effect of high pressure on the PL properties of MIL-140A(acetone).


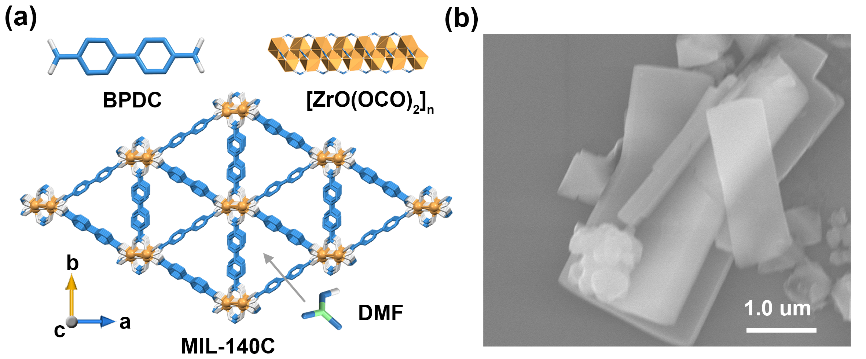


**Fig. S14** (a) Crystal structure of MIL-140C NCs at ambient conditions (H atoms are omitted for clarity). (b) Scanning electron microscope diagrams of MIL-140C NCs at ambient conditions.


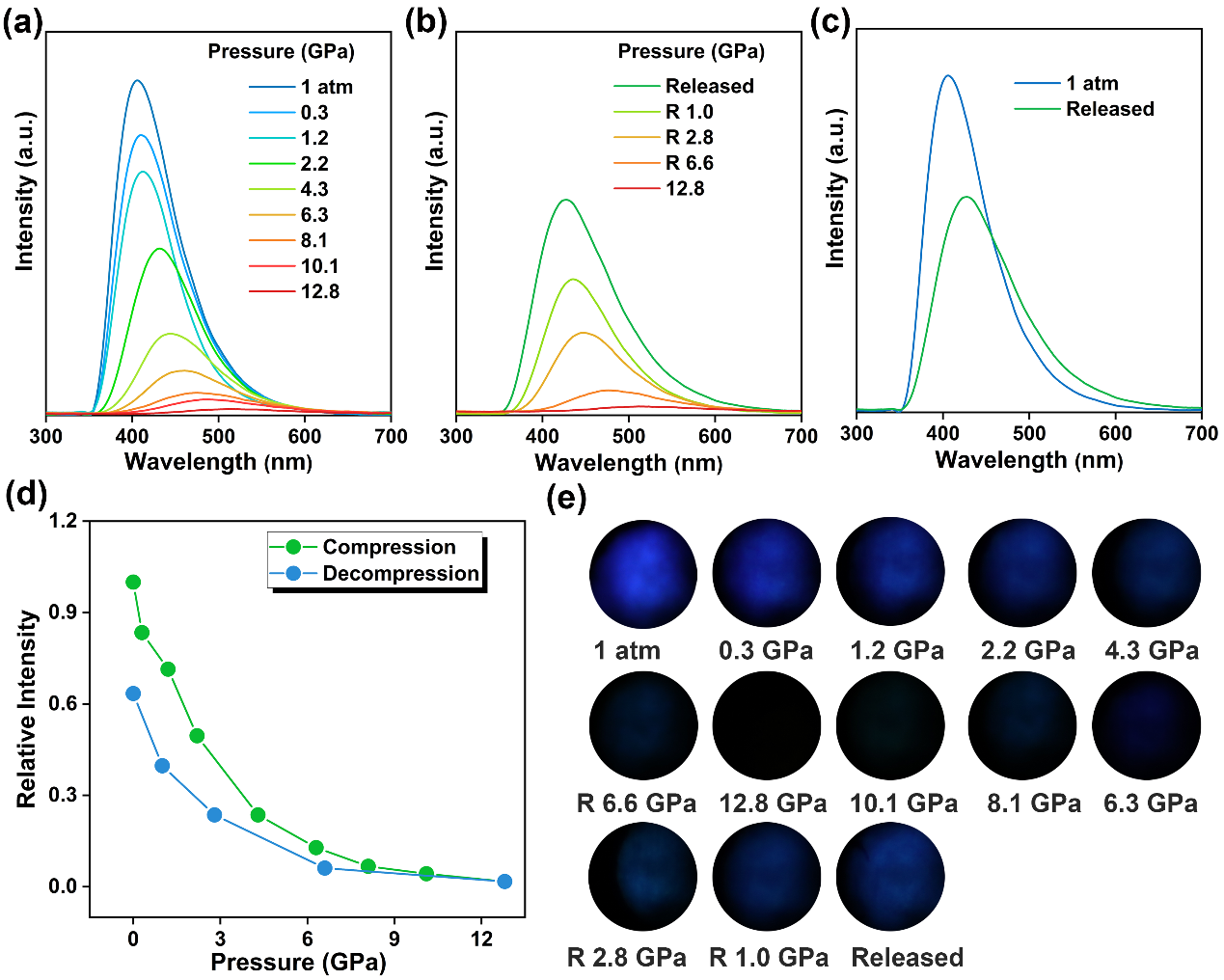


Fig. S15 (a-c) *In situ* high-pressure PL spectra of MIL-140C NCs under 355 nm laser excitation. (d) Evolution of emission intensity with pressure. (e) Corresponding PL photographs at different pressures.


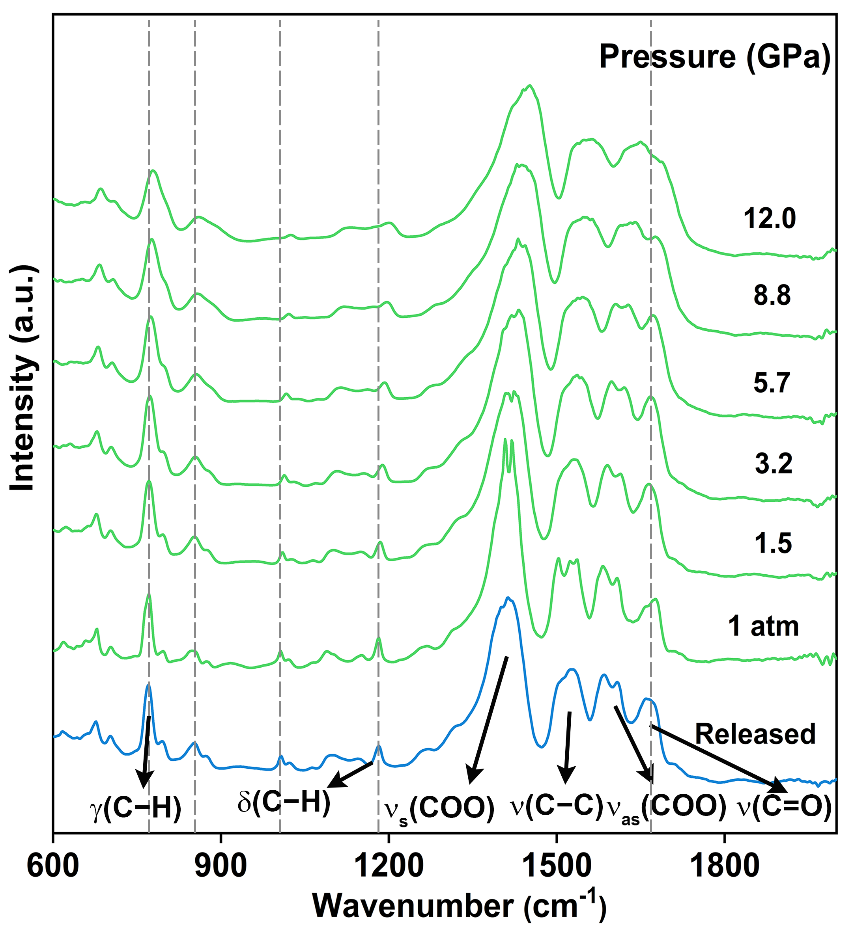


Fig. S16 *In situ* high-pressure IR absorption spectra for MIL-140C NCs in the region of 600-1900 cm^-1^. All IR absorption peaks shift to higher wavenumbers under pressure, accompanied by peak broadening. This indicates that MIL-140C undergoes structural contraction and a certain degree of structural distortion under pressure.


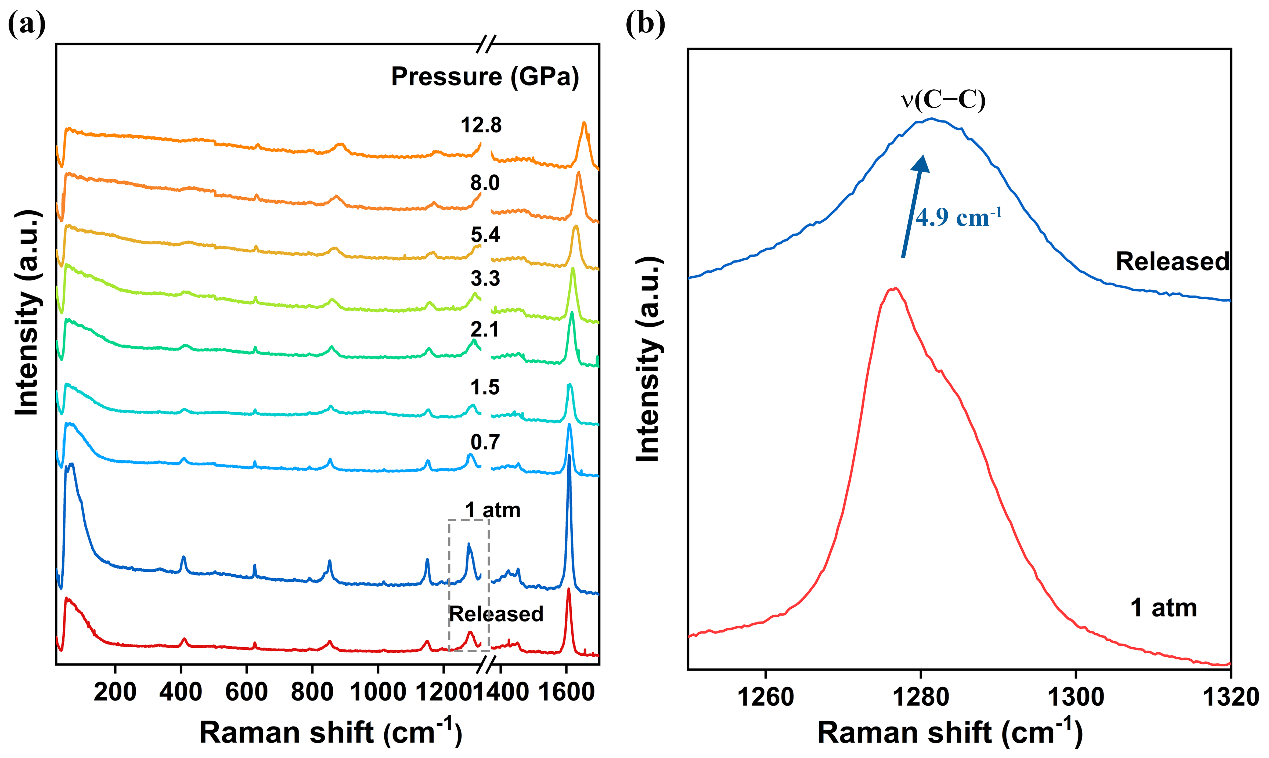


Fig. S17 (a) *In situ* high-pressure Raman scattering spectra of MIL-140C NCs in the region of 20-1700 cm^-1^. (b) The amplified Raman spectra before and after pressure treatment in the region of ν(C−C).

S3. Supplementary Tables

Table S1 The cell parameters of MIL-140A NCs based on GSAS refinements of the ADXRD before and after pressure treatment.

| Pressure | Space Group | Cell Length of *a* (Å) | Cell Length of *b* (Å) | Cell Length of *c* (Å) | Cell Angle of *α andγ* (°) | Cell Angle of *β* (°) | Cell Volume (Å^3^) |
| --- | --- | --- | --- | --- | --- | --- | --- |
| 1 atm | *C* 2*/c* | 25.59 | 11.20 | 7.82 | 90 | 112.5 | 2070.16 |
| Released | *C* 2*/c* | 25.28 | 11.20 | 7.83 | 90 | 112.6 | 2046.53 |

Table S2 Recognition of IR peaks in MIL-140A NCs [S4-9].

| Wavenumber(cm^-1^) | Description of the mode |
| --- | --- |
| 742 | Out-of-plane bending vibrations of aromatic C−H; γ(C−H) |
| 822 | Out-of-plane bending vibrations of aromatic C−H; γ(C−H) |
| 1017 | In-plane C-H bending vibrations of aromatic C−H; δ(C−H) |
| 1157 | In-plane C-H bending vibrations of aromatic C−H; δ(C−H) |
| 1375-1440 | Symmetric stretching vibrations of carboxyl group; ν_s_(O−C−O) |
| 1501 | Stretching vibrations of the benzene ring; ν(C−C) |
| 1532-1601 | Asymmetric stretching vibrations of carboxyl group; ν_as_(O−C−O) |
| 1665 | Stretching vibrations of the C=O in DMF; ν(C=O) |

Table S3 PLQYs of MIL-140A NCs before and after pressure treatment.

| Pressure | PL integral intensity  （a.u.） | | Cell  Volume  (Å^3^) | Absorbance | Refractive Index n | PLQY |
| --- | --- | --- | --- | --- | --- | --- |
| 1 atm | 23040 | 2070.16 | | 0.794 | 1.440 | 0.068 |
| Released | 242146 | 2046.53 | | 0.826 | 1.446 | 0.692 |

Table S4 Recognition of IR peaks in vacuum-dried MIL-140A NCs [S4-9].

| Wavenumber(cm^-1^) | Description of the mode |
| --- | --- |
| 742 | Out-of-plane bending vibrations of aromatic C−H; γ(C−H) |
| 825 | Out-of-plane bending vibrations of aromatic C−H; γ(C−H) |
| 1018 | In-plane C-H bending vibrations of aromatic C−H; δ(C−H) |
| 1158 | In-plane C-H bending vibrations of aromatic C−H; δ(C−H) |
| 1379-1442 | Symmetric stretching vibrations of carboxyl group; ν_s_(O−C−O) |
| 1503 | Stretching vibrations of the benzene ring; ν(C−C) |
| 1529-1574 | Asymmetric stretching vibrations of carboxyl group; ν_as_(O−C−O) |

Table S5 Recognition of IR peaks in MIL-140C NCs [S10-13].

| Wavenumber(cm^-1^) | Description of the mode |
| --- | --- |
| 770 | Out-of-plane bending vibrations of aromatic C−H; γ(C−H) |
| 854 | Out-of-plane bending vibrations of aromatic C−H; γ(C−H) |
| 1007 | In-plane C-H bending vibrations of aromatic C−H; δ(C−H) |
| 1182 | In-plane C-H bending vibrations of aromatic C−H; δ(C−H) |
| 1376-1440 | Symmetric stretching vibrations of carboxyl group; ν_s_(O−C−O) |
| 1521 | Stretching vibrations of the benzene ring; ν(C−C) |
| 1495-1547 | Asymmetric stretching vibrations of carboxyl group; ν_as_(O−C−O) |
| 1569-1615 | Asymmetric stretching vibrations of carboxyl group; ν_as_(O−C−O) |
| 1666 | Stretching vibrations of the C=O in DMF; ν(C=O) |

Supplementary References

[S1] J. Alfuth, B. Zadykowicz, B. Wicher, K. Kazimierczuk, T. Połoński et al., Cooperativity of halogen- and chalcogen-bonding interactions in the self-assembly of 4-iodoethynyl- and 4,7-bis(iodoethynyl)benzo-2,1,3-chalcogenadiazoles: crystal structures, hirshfeld surface analyses, and crystal lattice energy calculations. Cryst. Growth Des. 22(2), 1299–1311 (2022). https://doi.org/10.1021/acs.cgd.1c01266

[S2] Y. Wang, S. Guo, H. Luo, C. Zhou, H. Lin et al., Reaching 90% photoluminescence quantum yield in one-dimensional metal halide C_4_N_2_H_14_PbBr_4_ by pressure-suppressed nonradiative loss. J. Am. Chem. Soc. 142(37), 16001–16006 (2020). https://doi.org/10.1021/jacs.0c07166

[S3] W. Yin, C.-A. Tao, F. Wang, J. Huang, T. Qu et al., Tuning optical properties of MOF-based thin films by changing the ligands of MOFs. Sci. China Mater. 61(3), 391–400 (2018). https://doi.org/10.1007/s40843-017-9143-5

[S4] P.J. Jodłowski, G. Kurowski, K. Dymek, M. Oszajca, W. Piskorz et al., From crystal phase mixture to pure metal-organic frameworks-Tuning pore and structure properties. Ultrason. Sonochem. 95, 106377 (2023). https://doi.org/10.1016/j.ultsonch.2023.106377

[S5] V. Tzitzios, N. Kostoglou, M. Giannouri, G. Basina, C. Tampaxis et al., Solvothermal synthesis, nanostructural characterization and gas cryo-adsorption studies in a metal–organic framework (IRMOF-1) material. Int. J. Hydrog. Energy 42(37), 23899–23907 (2017). https://doi.org/10.1016/j.ijhydene.2017.04.059

[S6] E. Biemmi, T. Bein, N. Stock, Synthesis and characterization of a new metal organic framework structure with a 2D porous system: (H_2_NEt_2_)_2_[Zn_3_(BDC)_4_]⋅3DEF. Solid State Sci. 8(3–4), 363–370 (2006). https://doi.org/10.1016/j.solidstatesciences.2006.02.025

[S7] R. Chen, L. Cheng, J. Liu, Y. Wang, W. Ge et al., Toward high-performance CO_2_-to-C2 electroreduction via linker tuning on MOF-derived catalysts. Small 18(18), 2200720 (2022). https://doi.org/10.1002/smll.202200720

[S8] R. Dutta, M.N. Rao, A. Kumar, Investigation of ionic liquid interaction with ZnBDC-metal organic framework through scanning EXAFS and inelastic neutron scattering. Sci. Rep. 9(1), 14741 (2019). https://doi.org/10.1038/s41598-019-51344-0

[S9] A. Ehsani, S. Nejatbakhsh, A.M. Soodmand, M.E. Farshchi, H. Aghdasinia, High-performance catalytic reduction of 4-nitrophenol to 4-aminophenol using M-BDC (M = Ag, Co, Cr, Mn, and Zr) metal-organic frameworks. Environ. Res. 227, 115736 (2023). https://doi.org/10.1016/j.envres.2023.115736

[S10] V. Guillerm, F. Ragon, M. Dan-Hardi, T. Devic, M. Vishnuvarthan et al., A series of isoreticular, highly stable, porous zirconium oxide based metal-organic frameworks. Angew. Chem. Int. Ed. 51(37), 9267–9271 (2012). https://doi.org/10.1002/anie.201204806

[S11] Q.-R. Fang, G.-S. Zhu, Z. Jin, Y.-Y. Ji, J.-W. Ye et al., Mesoporous metal–organic framework with rare etb topology for hydrogen storage and dye assembly. Angew. Chem. Int. Ed. 46(35), 6638–6642 (2007). https://doi.org/10.1002/anie.200700537

[S12] S. Chavan, J.G. Vitillo, D. Gianolio, O. Zavorotynska, B. Civalleri et al., H_2_ storage in isostructural UiO-67 and UiO-66 MOFs. Phys. Chem. Chem. Phys. 14(5), 1614–1626 (2012). https://doi.org/10.1039/c1cp23434j

[S13] A.R.K. Chatenever, L.R. Warne, J.E. Matsuoka, S.J. Wang, E.W. Reinheimer et al., Isomorphous lanthanide metal–organic frameworks based on biphenyldicarboxylate: synthesis, structure, and photoluminescent properties. Cryst. Growth Des. 19(8), 4854–4859 (2019). https://doi.org/10.1021/acs.cgd.9b00840
